# ***Streptococcus suis* in Humans, Thailan**d

**DOI:** 10.3201/eid1401.070568

**Published:** 2008-01

**Authors:** Daisuke Takamatsu, Korawan Wongsawan, Makoto Osaki, Hiroto Nishino, Tomono Ishiji, Prasit Tharavichitkul, Banyong Khantawa, Achara Fongcom, Shinji Takai, Tsutomu Sekizaki

**Affiliations:** * National Institute of Animal Health, Tsukuba, Ibaraki, Japan; † Chiang Mai University, Chiang Mai, Thailand; ‡ Northern District Livestock Health and Hygiene Office, Mito, Ibaraki, Japan; § Seibu Livestock Hygiene Service Center, Tonami, Toyama, Japan; ¶ Lamphun Provincial Hospital, Lamphun, Thailand; # Kitasato University, Towada, Aomori, Japan; ** Gifu University, Gifu, Gifu, Japan

**Keywords:** *Streptococcus suis*, Thailand, human, swine, infection, bacterial typing techniques, letter

## Abstract

*Streptococcus suis* in Humans, Thailand

**To the Editor:** *Streptococcus suis* is an important zoonotic pathogen for swine and humans. Among 33 serotypes, serotype 2 is more frequently isolated from diseased pigs than other serotypes (*1*). However, not all serotype 2 strains are virulent, and degree of virulence varies among strains (*2*). Previous studies have reported several *S. suis* putative virulence factors, including the polysaccharide capsule, the muramidase-released protein, the extracellular factor, and suilysin (*3**–**5*). Some of these factors have been used as virulence-associated markers, and the association of the factors of *S. suis* isolates with virulence or clinical background has been suggested in Europe (*2*,*5*). However, because many virulent isolates lacking these factors have also been isolated from clinical cases in Canada (*6*), they cannot be used as virulence markers in North America.

Recent analysis of *S. suis* isolates by multilocus sequence typing (MLST) suggested the association of some clonal groups with particular clinical manifestations. That is, most invasive isolates belonged to the sequence type (ST) 1 complex, while the ST27 and ST87 complexes were found to include a higher proportion of lung isolates (*7*). Although *S. suis* has been prevalent worldwide, the geographic location of the isolates used so far was mainly Europe, North America, and East Asia (*7**–**9*). Moreover, the clonal association with virulence of *S. suis* has been discussed mainly on the basis of clinical and experimental data in swine (*7*). In this report, to broaden understanding of the population structure of *S. suis* as a zoonotic agent, we characterize 20 *S. suis* isolates (Table) recovered from humans in Thailand in 1998–2002.

**Table:** Epidemiologic data of *Streptococcus suis* isolates from patients in Thailand, 1998–2002*

Isolate no.†	Year of isolation	Site of isolation	Virulence-associated genes‡§	Serotype	Diseases and symptoms	ST (ST complex)
MNCM01	2000	Blood	*cps2J*+/*sly*+/*epf*+/*mrp*+	2¶	Endocarditis	1 (1)
MNCM06	2000	Blood, CSF	*cps2J*+/*sly*+/*epf*+/*mrp*+	2	Neck stiffness, deafness (meningitis)	1 (1)
MNCM16	2000	CSF	*cps2J*+/*sly*+/*epf*+/*mrp*+	2	Neck stiffness (meningitis)	1 (1)
MNCM07	2000	Blood, CSF	*cps1J*+/*sly*+/epf*/*mrp*^S^+	14	Neck stiffness (meningitis), death	11 (1)
MNCM04	2000	Blood	*cps2J*+/*sly–*/*epf–*/mrp**+	2	Neck stiffness, deafness (meningitis)	25 (27)#
MNCM10	2000	Blood	*cps2J*+/*sly–*/*epf–*/mrp**+	2	Septicemia	25 (27)#
MNCM24	2001	Blood	*cps2J*+/*sly–*/*epf–*/mrp**+	2¶	Endocarditis	25 (27)#
MNCM26	2001	Blood	*cps2J*+/*sly–*/*epf–*/mrp**+	2	Endocarditis, deafness (meningitis)	25 (27)#
MNCM51	2002	Blood	*cps2J*+/*sly–*/*epf–*/mrp**+	2	Septicemia, diarrhea, death	25 (27)#
MNCM55	2002	Blood	*cps2J*+/*sly–*/*epf–*/mrp**+	2	Septic shock, death	25 (27)#
LPH4	2001	Blood	*cps2J*+/*sly–*/*epf–*/mrp**+	2	Septicemia, diarrhea	25 (27)#
LPH12	2002	Blood	*cps2J*+/*sly–*/*epf–*/mrp**+	2	Septic shock, death	25 (27)#
MNCM43	2002	Blood	*cps2J*+/*sly–*/*epf–*/*mrp*+	2	Endocarditis	28 (27)
MNCM21	1998	CSF	*cps2J*+/*sly*+/*epf–*/*mrp–*	2	Meningitis	101 (27)#
MNCM25	2001	Blood	*cps2J*+/*sly–*/*epf–*/mrp**+	2	Neck stiffness (meningitis), diarrhea, death	102 (27)#
MNCM54	2002	Blood	*cps2J*+/*sly–*/*epf–*/–mrp**+	2	Neck stiffness (meningitis), diarrhea	102 (27)#
MNCM33	2002	Blood, CSF	*cps2J*+/*sly–*/*epf–*/mrp**+	2	Neck stiffness (meningitis)	103 (27)#
LPH3	2001	Blood	*cps2J*+/*sly–*/*epf–*/mrp**+	2	Meningitis	103 (27)#
LPH5	2001	Blood	*cps2J*+/*sly–*/*epf–*/mrp**+	2	Septicemia	103 (27)#
MNCM50	2002	Blood	*cps2J*+/*sly*+/*epf–*/*mrp–*	2	Pulmonary edema, death	104 (27)#

*ST, sequence type; CSF, cerebrospinal fluid. †Isolates with MNCM number and LPH number were isolated from patients at Maharaj Nakorn Chiang Mai Hospital and Lamphun Hospital, Thailand, respectively. ‡Virulence-associated gene profiling was done as described previously (*10*). *cps1J* and *cps2J*, serotype 1 (and 14) and 2 (and 1/2) specific genes, respectively, involved in the capsular biosynthesis; *sly*, suilysin gene; *epf*, extracellular factor gene; *mrp*, muraminidase-released protein gene; +, positive; –, negative. §epf*, an *epf* variant that produces an ≈3,000-bp fragment by PCR with primers described previously (*10*); mrp** and *mrpS*, *mrp* variants that produce ≈1,800-bp and ≈750-bp fragments, respectively, by PCR with primers described previously (*10*). ¶Coagglutination reaction using anti-serotype 2 serum was weak. #ST25, ST101, ST102, ST103, and ST104 belong to the ST27 complex, only with a less-stringent approach that defines an ST complex by sharing of alleles at >5 of the 7 loci.

Serotyping by coagglutination tests showed that 19 of the 20 isolates belonged to serotype 2, while the remaining 1 (MNCM07) was serotype 14. MLST analysis resolved the 20 isolates into 8 STs (Table). By using eBURST (http://eburst.mlst.net), we assigned 4 isolates (MNCM01, MNCM06, MNCM07, and MNCM16) from 1 case of endocarditis and 3 cases of meningitis to the ST1 complex. The remaining isolates were assigned to the ST27 complex with a less-stringent group definition (Table), although ST101 (MNCM21) and ST104 (MNCM50) shared only 2 alleles with ST27 and were incorporated into this complex by a chaining effect. Regarding the clinical cases from which the ST27 complex isolates were recovered, the patients had meningitis, endocarditis, septicemia, septic shock, diarrhea, and respiratory involvement. The 2 ST complexes both contained isolates from deceased patients (Table).

All the isolates assigned to the ST1 complex were positive for the suilysin gene *sly*, the extracellular factor gene *epf* or its variant, and the muramidase-released protein gene *mrp* or its variant. With the exception of MNCM21 and MNCM50, which had only *sly*, all isolates classified into the ST27 complex were negative for *sly* and *epf* but positive for *mrp* or its variant. These results showed the congruence between STs and the virulence-associated gene profiles and further support the usefulness of MLST for epidemiologic studies of *S. suis*.

Of the 3 major clonal complexes identified so far in *S. suis* (ST1, ST27, and ST87), the ST1 complex particularly attracts considerable public attention as a clonal group that may have the potential for a higher degree of virulence than the others (*7*), and most (96%) of the human isolates investigated so far, including ST7 isolates, which caused the largest outbreak in China, belong to the ST1 complex (*7**–**9*). In this study, although no ST7 isolate was found, 4 isolates were assigned to the ST1 complex. This further confirmed the gravity of the ST1 complex not only for swine industries but also for public health.

In contrast to the ST1 complex, only 4 human clinical isolates have so far been reported to belong to the ST27 complex. Three of the 4 are isolates from Canada that belong to ST25 (*7*). The remaining 1 is from Japan and assigned to ST28 (*8*). Unlike in previous reports, 80% of the human clinical isolates (16 isolates) characterized in this study were assigned to the ST27 complex. Although previous studies suggested that members of the ST27 complex may have lower potential to cause invasive diseases in swine (*7*), all the isolates were isolated from blood or cerebrospinal fluid of the patients, suggesting a high degree of invasiveness (Table). Because it is unknown whether the ST27 complex is also dominant among isolates from diseased pigs in Thailand, future surveillance will be necessary to know the situation in pigs. However, our data indicate that the ST27 complex is another clonal group that should be assessed for its importance for human infection. Because *mrp*, *epf*, and *sly* are not appropriate as virulence markers for the ST27 complex members, development of novel virulence markers will be needed for efficient discrimination of *S. suis* strains virulent for humans.

## References

[1] Staats JJ, Feder I, Okwumabua O, Chengappa MM. *Streptococcus suis*: past and present. Vet Res Commun. 1997;21:381–407. 10.1023/A:1005870317757 9266659

[2] Vecht U, Wisselink HJ, van Dijk JE, Smith HE. Virulence of *Streptococcus suis* type 2 strains in newborn germfree pigs depends on phenotype. Infect Immun. 1992;60:550–6. 1730489 10.1128/iai.60.2.550-556.1992 PMC257663

[3] Jacobs AAC, Loeffen PLW, van den Berg AJG, Storm PK. Identification, purification, and characterization of a thiol-activated hemolysin (suilysin) of *Streptococcus suis.* Infect Immun. 1994;62:1742–8. 8168935 10.1093/benz/9780199773787.article.b00034458 PMC186398

[4] Smith HE, Damman M, van der Velde J, Wagenaar F, Wisselink HJ, Stockhofe-Zurwieden N, Identification and characterization of the *cps* locus of *Streptococcus suis* serotype 2: the capsule protects against phagocytosis and is an important virulence factor. Infect Immun. 1999;67:1750–6. 10085014 10.1128/iai.67.4.1750-1756.1999 PMC96524

[5] Vecht U, Wisselink HJ, Jellema ML, Smith HE. Identification of two proteins associated with virulence of *Streptococcus suis* type 2. Infect Immun. 1991;59:3156–62. 1879937 10.1128/iai.59.9.3156-3162.1991 PMC258147

[6] Gottschalk M, Lebrun A, Wisselink H, Dubreuil JD, Smith H, Vecht U. Production of virulence-related proteins by Canadian strains of *Streptococcus suis* capsular type 2. Can J Vet Res. 1998;62:75–9. 9442945 PMC1189447

[7] King SJ, Leigh JA, Heath PJ, Luque I, Tarradas C, Dowson CG, Development of a multilocus sequence typing scheme for the pig pathogen *Streptococcus suis*: identification of virulent clones and potential capsular serotype exchange. J Clin Microbiol. 2002;40:3671–80. 10.1128/JCM.40.10.3671-3680.2002 12354864 PMC130843

[8] Chang B, Wada A, Ikebe T, Ohnishi M, Mita K, Endo M, Characteritics of *Streptococcus suis* isolated from patients in Japan. Jpn J Infect Dis. 2006;59:397–9. 17186962

[9] Ye C, Zhu X, Jing H, Du H, Segura M, Zheng H, *Streptococcus suis* sequence type 7 outbreak, Sichuan, China. Emerg Infect Dis. 2006;12:1203–8. 16965698 10.3201/eid1208.060232 PMC3291228

[10] Silva LMG, Baums CG, Rehm T, Wisselink HJ, Goethe R, Valentin-Weigand P. Virulence-associated gene profiling of *Streptococcus suis* isolates by PCR. Vet Microbiol. 2006;115:117–27. 10.1016/j.vetmic.2005.12.013 16431041

