# ***Streptococcus suis* in Humans, Thailan**d

**DOI:** 10.3201/eid0805.

**Published:** 2002-05

## Correction, Vol. 8, No. 2

In the article “Epidemiology of *Burkholderia cepacia* Complex in Patients with Cystic Fibrosis, Canada” by David P. Speert et al., an error was made in calculations for Table 2 on page 184. The corrected table appears below and online at http://www.cdc.gov/ncidod/eid/vol8no2/01-0163.htm. In addition, the corrected percentages appear in two sentences from the results section on page 183, as follows: Most isolates (82.5%) were from genomovar III and included all strains that clustered in individual centers and appeared to be transmitted from patient to patient. Approximately 10% of infected patients were infected with *B. multivorans* (genomovar II), but there was little evidence among these isolates of genotypic clustering as determined by RAPD and PFGE.

**Table 2:** Genomovar or species of *Burkholderia cepacia* complex or phenotypically similar isolates from cystic fibrosis patients in Canada

Species or genomovar	No. of patients infected with species or genomovar^a^	Percentage of patients (%)
Genomovar I	1	0.2
*Burkholderia multivorans* (genomovar II)	43	9.6
Genomovar III	369	82.5
*Burkholderia stabilis* (genomovar IV)	17	3.8
*Burkholderia vietnamiensis* (genomovar V)	7	1.6
*Burkholderia cepacia* complex (not genomovar I-VII)	8	1.8
*Burkholderia fungorum*	1	0.2
*Burkholderia gladioli*	5	1.1
*Ralstonia pickettii*	5	1.1
*Pandoraea* spp.	5	1.1
Total	461^a^	

^a^Some patients were counted twice if two or more different strains were recovered; therefore, the percentage of patients is based on a denominator of 447.

We regret any confusion this error may have caused.

